# Congestive Heart Failure Effects on Atrial Fibroblast Phenotype: Differences between Freshly-Isolated and Cultured Cells

**DOI:** 10.1371/journal.pone.0052032

**Published:** 2012-12-14

**Authors:** Kristin Dawson, Chia-Tung Wu, Xiao Yan Qi, Stanley Nattel

**Affiliations:** 1 Research Center, Montreal Heart Institute, Montreal, Quebec, Canada; 2 Department of Pharmacology and Therapeutics, McGill University, Montreal, Quebec, Canada; 3 Department of Medicine, Montreal Heart Institute and Université de Montréal, Montreal, Quebec, Canada; 4 Chang-Gung Memorial Hospital and University Taoyuan, Taoyuan, Taiwan, Republic of China; University of Bergen, Norway

## Abstract

**Introduction:**

Fibroblasts are important in the atrial fibrillation (AF) substrate resulting from congestive heart failure (CHF). We previously noted changes in *in vivo* indices of fibroblast function in a CHF dog model, but could not detect changes in isolated cells. This study assessed CHF-induced changes in the phenotype of fibroblasts freshly isolated from control versus CHF dogs, and examined effects of cell culture on these differences.

**Methods/Results:**

Left-atrial fibroblasts were isolated from control and CHF dogs (ventricular tachypacing 240 bpm×2 weeks). Freshly-isolated fibroblasts were compared to fibroblasts in primary culture. Extracellular-matrix (ECM) gene-expression was assessed by qPCR, protein by Western blot, fibroblast morphology with immunocytochemistry, and K^+^-current with patch-clamp. Freshly-isolated CHF fibroblasts had increased expression-levels of collagen-1 (10-fold), collagen-3 (5-fold), and fibronectin-1 (3-fold) vs. control, along with increased cell diameter (13.4±0.4 µm vs control 8.4±0.3 µm) and cell spreading (shape factor 0.81±0.02 vs. control 0.87±0.02), consistent with an activated phenotype. Freshly-isolated control fibroblasts displayed robust tetraethylammonium (TEA)-sensitive K^+^-currents that were strongly downregulated in CHF. The TEA-sensitive K^+^-current differences between control and CHF fibroblasts were attenuated after 2-day culture and eliminated after 7 days. Similarly, cell-culture eliminated the ECM protein-expression and shape differences between control and CHF fibroblasts.

**Conclusions:**

Freshly-isolated CHF and control atrial fibroblasts display distinct ECM-gene and morphological differences consistent with *in vivo* pathology. Culture for as little as 48 hours activates fibroblasts and obscures the effects of CHF. These results demonstrate potentially-important atrial-fibroblast phenotype changes in CHF and emphasize the need for caution in relating properties of cultured fibroblasts to *in vivo* systems.

## Introduction

Fibroblasts are the most common cells in connective tissue and are responsible for maintaining the extracellular matrix (ECM) throughout the body [1]. Because their properties are adapted to specific tissue structures and functions, fibroblast populations are heterogeneous across tissue types, within tissue subtypes, among species, and in disease conditions [2]–[9]. Under cardiac disease conditions (such as congestive heart failure (CHF)) or during wound repair (after myocardial infarction (MI)), fibroblasts differentiate into an activated myofibroblast phenotype, which is much less expressed in healthy adult tissue. Myofibroblasts exhibit migratory, proliferative, and secretory properties, resulting in the accumulation of ECM and the development of tissue fibrosis [5]. Celiac-patient fibroblasts display a different *in vitro* morphology compared to non-celiac subject fibroblasts [6]. Fibroblasts from systemic sclerosis patients also react differently to Imatinib treatment compared to controls [7]. Even fibroblasts from the same organ can behave differently in different variants of similar diseases. For example skin fibroblasts from sporadic and familial Alzheimer’s patients show different ECM expression-patterns and react differently to fibroblast growth factor [8]. Fibroblasts from rheumatoid arthritis patients displayed a different gene expression pattern compared to fibroblasts from osteoarthritis patients [9].

In a variety of cardiac disease models of congestive heart failure (CHF), an extensive atrial fibrotic response occurs with comparatively little ventricular fibrosis [10]. This difference is reflected in ECM gene expression patterns, with CHF atrial tissue displaying robust increases in mRNA expression of collagen1A1 (COL1A1), collagen3A1 (COL3A1), and fibronectin (FN1) compared to control, whereas CHF ventricular tissue does not [11]. As fibroblasts are the main cell type responsible for secreting ECM components, atrial and ventricular fibroblasts behave differently in response to the same disease setting (CHF). CHF can be induced experimentally by ventricular tachypacing (VTP) at a high rate (240-bpm for 2 weeks), mimicking the clinical condition of tachycardiomyopathy [3], [12].

Much of our knowledge about fibroblast behavior has been derived from data obtained within *in vitro* cell culture systems. Cell culture is a convenient way to purify cardiac fibroblasts from common but poorly/non-proliferating cell-types like cardiomyocytes. In addition, cell culture allows for the observation of cellular responses to specifically defined stimuli [13]. Different types of fibroblasts have been extensively characterized in culture [2], [3], [14]; however, the degree to which cell culture itself alters fibroblast phenotype and obscures the changes induced by cardiac disease is not well understood. Atrial and ventricular fibroblasts have innate differences that are maintained in culture, but tend to decrease over time [3]. In a previous study, we noted differences between *in vivo* indices of CHF and control fibroblast function (such as gene expression) [3]; however, under cell culture conditions these differences were not seen *in vitro*. This observation led us to speculate that the changes in atrial fibroblast phenotype caused by profibrotic disease conditions *in vivo* might be obscured by the changes occurring *in vitro*. Therefore, we designed the present study to: 1) determine the changes in atrial fibroblast phenotype resulting from CHF as reflected by freshly-isolated fibroblasts and 2) compare selected remodeling-related changes in freshly-isolated versus cultured canine atrial fibroblasts. Specifically, we examined changes in ECM gene expression, cell histomorphometry, and tetraethylammonium (TEA)-sensitive ion-current expression. Our results suggest that cell culture alters the phenotype of cardiac fibroblasts in ways that mimic remodeling effects, and consequently has the capacity to obscure the effects of remodeling. These findings urge caution with the use of cultured fibroblast systems in studying the effects of cardiac remodeling, and also in extrapolating behaviours observed *in vitro* to the *in vivo* condition.

## Methods

### Animal Model

All animal protocols were approved by the Ethics Committee of the Montreal Heart Institute and followed National Institutes of Health guidelines. Thirty-three adult male mongrel dogs (25–35 kg) were used: control (18 dogs) and 2-week VTP-induced CHF (15 dogs). CHF-dogs were anesthetized under diazepam (0.25 mg/kg IV)/ketamine (5.0 mg/kg IV)/halothane (1% to 2% PI) anaesthesia so that two leads could be inserted into the right-ventricular apex (via left jugular vein under fluoroscopy) and connected to a pacemaker (St. Jude Medical, St. Paul, MN) implanted subcutaneously in the neck. Following twenty-four hours post-operative recovery time, ventricular-pacing was initiated at 240 bpm. CHF was confirmed by signs and *in vivo* hemodynamic findings. After the pacing period was completed, dogs were anesthetized with morphine (2 mg/kg SC) and α-chloralose (120 mg/kg IV, followed by 29.25 mg/kg/h) and ventilated mechanically. Hearts were removed via median thoracotomy and immediately immersed in oxygenated Tyrode solution containing (in mM): NaCl 136, KCl 5.4, MgCl_2_ 1, CaCl_2_ 2, NaH_2_PO_4_ 0.33, HEPES 5 and dextrose 10, pH 7.35 (NaOH). Atrial tissue samples were immediately frozen in liquid-N_2_ and stored at −80°C and tissue was subjected to enzymatic digestion as described below for cell isolation.

### Fibroblast Isolation and Culture

The left circumflex coronary artery was cannulated and all leaking branches were ligated, followed by perfusion with Ca^2+^-free Tyrode solution for 10 minutes. The preparation was then perfused at 10 mL/min with Ca^2+^-free Tyrode solution containing type II collagenase (150 U/mL) and albumin (0.1%) for one hour. The harvested cells were collected in either DMEM medium (for culture) or Tyrode solution (for patch-clamp). Cells in DMEM were dispersed by gentle trituration with a pipette. Filtration (500-µm nanomesh) was used to remove debris and cells were centrifuged at 800 rpm for 5 minutes to pellet cardiomyocytes. The supernatant was collected and filtered through 20 µm nanomesh and centrifuged at 2,000 rpm for 10 minutes to pellet fibroblasts. Pelleted, freshly isolated fibroblasts were then separated; one half was immediately frozen in liquid-N_2_ and stored for RNA extraction, and the remaining cells were cultured. Cells were cultured for varying lengths of time (24 hours, 48 hours, 4 days, 7 days, 15 days) on plastic 25 cm^2^ culture dishes (Sarstedt, 83.1810.002). Cells were also cultured for 48 hours on 6-well plastic (Corning Inc., 3605) or 6-well collagen-I coated plastic plates (Millipore, PICL06P05). Cells were incubated at 5% CO_2_/95%-humidified air (37°C) in DMEM supplemented with 10% fetal bovine serum and 1% penicillin/streptomycin. A medium change was performed 1–2 hours after plating to remove any dead cells and debris. For the cell culture time-course experiments, medium was changed 24 hours prior to cell-harvesting, and at the time of cell-harvesting medium was immediately frozen in liquid-N_2_ for subsequent Western blot analysis. After culture, cells were detached with 0.25% trypsin-EDTA, counted, and then pelleted by centrifugation followed by immediate freezing in liquid-N_2_ for RNA extraction.

### Western Blots

Pulmonary artery, digested tissue, cardiomyocyte fraction, and freshly isolated fibroblasts were protein extracted and quantified by the Bradford assay. Protein samples were loaded at 30 µg/lane and separated on a 6% SDS-PAGE gel (smoothelin), 8% SDS-PAGE gel (vimentin), or 12% SDS-PAGE gel (caveolin 3) by electrophoresis and transferred to polyvinylidenedifluoride (PVDF) membranes. Membranes were blocked for 1 hr at room temperature with milk and incubated with mouse anti-smoothelin (1/1,000 Millipore MAB3242), mouse anti-vimentin (1/1,000 Santa Cruz – sc-32322), mouse anti-caveolin3 (1/2,000 BD Transduction Labs #610421) overnight, afterwards were incubated with mouse anti-GAPDH (1/10,000) for 1 hr. Membranes were washed and then incubated with anti-mouse (1/10,000 Jackson) horseradish peroxidase-conjugated secondary antibodies for 1 hr at room temperature. Signals were detected with chemiluminescence and quantified by Biorad Quantity One software. All proteins are expressed relative to GAPDH.

For ECM-protein detection, supernatant loading was adjusted based on cell number, with a maximum volume of 60 µL and minimum volume of 10 µL loaded. Samples were run on a 6% SDS-PAGE gel and separated by electrophoresis, then transferred to nitrocellulose membranes. Membranes were blocked overnight in 3% bovine serum albumin, then incubated with primary antibodies to collagen-I (1/20,000 MD Biosciences, MD20151) or fibronectin (1/1,000 Abcam, Ab6328) for 1 hour at room temperature. Membranes were washed and then incubated with anti-rabbit (1/10,000 Jackson for collagen) or anti-mouse (1/10,000 Jackson for fibronectin) horseradish peroxidase-conjugated secondary antibodies for 1 hour at room temperature. Signals were detected with chemiluminescence and quantified with Biorad Quantity One software. All proteins are expressed relative to cell number.

### Quantitative Polymerase Chain Reaction (qPCR)

RNA was extracted with Trizol (Ambion Carlsbad, CA) following the manufacturer’s protocol. RNA was dissolved in RNase free water and its concentration was determined by nanodrop. cDNA was synthesized from 1 µg RNA (or 0.5 µg for time-course experiments) with the use of a High Capacity cDNA Reverse Transcription Kit (Applied Biosystems Foster City, CA). qPCR was performed with TaqMan probes and primers (Applied BiosystemsFoster City, CA): COL3A1 (Assay ID: cf02631369_m1), FN1 (cf0269549_m1), B2M (cf02659077_m1). COL1A1 primers were custom-made (Forward primer: 5′ CCAAGAGGAGGGCCAAGAA; Reverse primer: 5′AGTACCTGAGGCCGTTCTGTA; Probe –5′ ACTGGTGGGATGTCTTC). Beta-2-microglobulin (B2M) was used as an internal standard for all genes, to which their expression values were normalized to. Each gene was detected in duplicate and analyzed with the delta-CT method.

### Immunofluorescence

Fibroblasts from isolated canine hearts were plated in FluoroDishes (World Precision Instruments Sarasorta, FL) and placed into the incubator for 1.5 hours (freshly isolated) or 48 hours (cultured). Fibroblasts were washed with medium and then fixed with 2% paraformaldehyde (in phosphate buffered saline (PBS) containing 1.37 M NaCl, 26.8 mM KCl, 42.3 mM hydrated Na_2_HPO_4_, 17.6 mMKH_2_PO_4_) for 15 minutes. Fibroblasts were then blocked and permeabilized with normal donkey serum containing Triton (10% in PBS) for 1 hour. Cells were incubated overnight with primary antibodies: anti-vimentin (1∶200, Santa Cruz SC-7557) and anti-αSMA (1∶400, Sigma A5228). After washing with PBS, cells were incubated for 1 hour with the secondary antibody: AlexaFluor 488-conjugated donkey anti-goat (1∶600) or AlexaFluor 555-conjugated donkey anti-mouse (1∶600) and 4',6-diamidino-2-phenylindole (DAPI, 1∶10,000, Invitrogen D3571, Ontario, Canada). Cells were washed, 1,4-diazabicyclo[2.2.2]octane (DABCO) 10 uL was added, glass cover slip was placed over the top of the cells, and the coverslip was sealed to the dish with nail polish. Confocal images were acquired with a Zeiss LSM 510 confocal microscope equipped with 40x/1.3 Plan-Apochromat oil with a Differential Interference Contrast (DIC) objective. The fluorochromes 4',6-diamidino-2-phenylindole (DAPI, a nuclear counterstain), Alexa 488, and Alexa 555 were excited and collected sequentially with lasers at 405 nm (420–480 nm), Argon 488 nm (505–530 nm), and HeNe 543 nm (560–615 nm) respectively. Quantification of shape factor was performed with the Zeiss LSM image browser to acquire perimeter and area of cells. The following formula was used to calculate shape factor (SF): SF  = 4πA/P^2^ with A = area and P = perimeter.

### Cellular Electrophysiology/Patch Clamp

Purified freshly isolated or cultured fibroblasts were superfused with Tyrode solution (containing 2 mMCaCl_2_) as the external solution. Borosilicate glass microelectrodes (tip resistance 5–10 MΩ) were filled with internal solution containing (in mM):GTP 0.1, K^+^-aspartate 110, KCl 20, MgCl_2_ 1, ATP-Mg 5, HEPES 10, Na_2_-phosphocreatine 5, and ethylene glycol tetraacetic acid(EGTA) 0.05; pH 7.4 (KOH). Whole-cell voltage clamp was performed and all current recordings were obtained at 35±1°C. Series resistance and cell capacitance were compensated. Mean fibroblast capacitance was 11±1.2 pF for freshly isolated control and 22±2.8 pF for freshly isolated CHF fibroblasts. Leak currents were not subtracted. Junction potentials between pipette and bath averaged -10.5 mV and were not corrected.

### Statistical Analysis

Results are presented as mean±SEM. Unpaired *t*-tests were used for two-group comparisons. For comparison of three groups or more for one variable a one-way ANOVA was used followed by a Dunnett’s test. For comparison of control and CHF in freshly isolated or 48 hr culture conditions a 2-way ANOVA was performed followed by Bonferroni post-hoc tests. A two-tailed *P*<0.05 was considered statistically-significant.

## Results

### In vivo Tissue vs. in vitro Cultured Fibroblast Collagen Gene Expression

**Figure 1:**
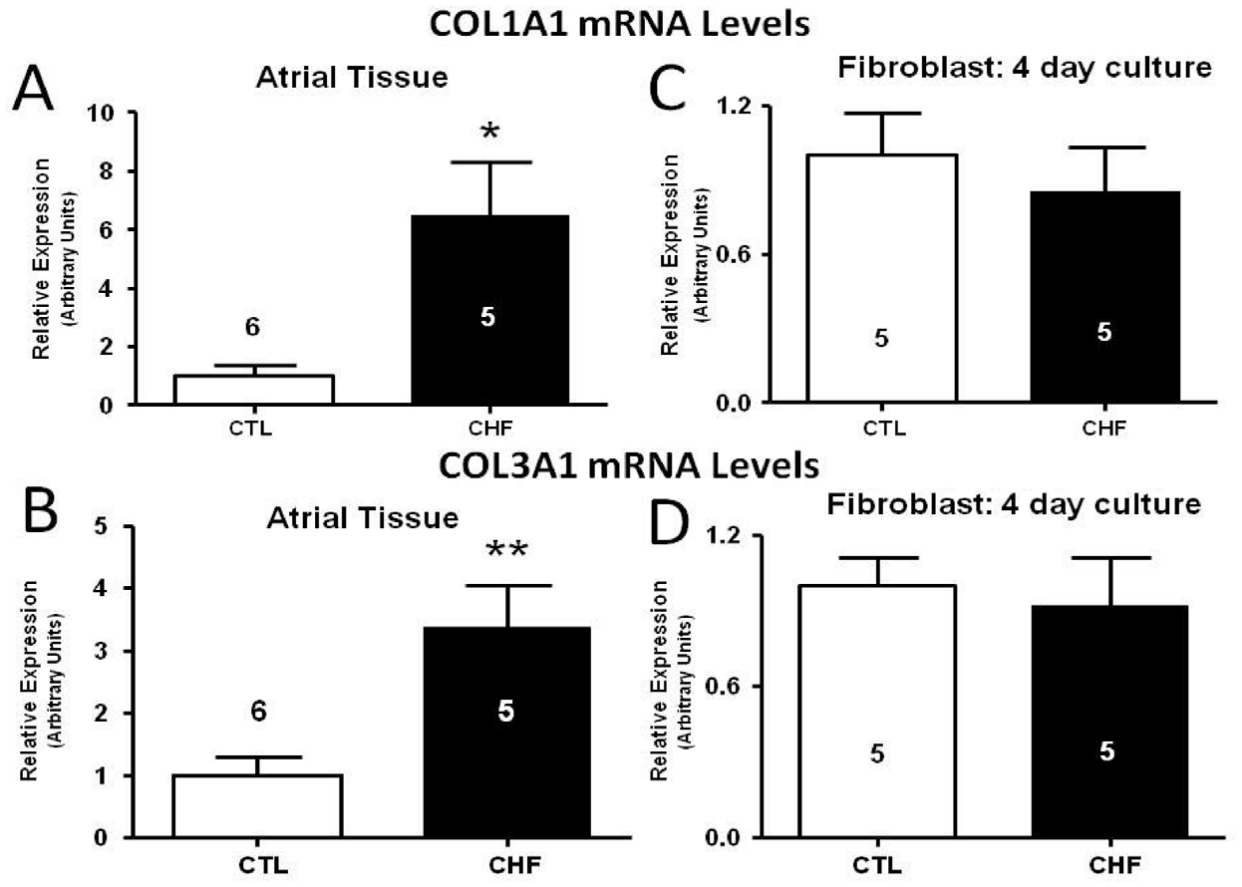
Gene expression of collagen I (COL1A1; top) and collagen III (COL3A1; bottom) in intact atrial tissue samples and in 4 day cultured atrial fibroblasts as measured by qPCR. (A) COL1A1 mRNA levels in atrial tissue. (B) COL3A1 mRNA levels in atrial tissue. (C) COL1A1 mRNA levels in atrial fibroblasts after culture. (D) COL1A1 mRNA levels in atrial fibroblasts after culture. **P<0.01, *P<0.05 by t-test, number of dogs indicated in figure.

We first confirmed the atrial ECM-gene expression phenotype by observing that atrial tissues from CHF dogs display approximately 6–8 fold increases in mRNA expression of collagen1A1 (COL1A1) as compared to control dogs (Figure 1A). Similarly, collagen3A1 (COL3A1) mRNA expression is about 3–4 fold higher in the atrial tissue of CHF dogs versus controls (Figure 1B). Fibroblasts were isolated and placed into culture in order to relate the differences in behaviour between CHF and control fibroblasts *in vitro* to the changes noted with *in vivo* tissues. In fibroblasts cultured for 4 days, mRNA expression of both COL1A1 and COL3A1 were indistinguishable between control and CHF fibroblasts (Figure 1C and 1D).

### Gene Expression in Freshly Isolated vs. Cultured Atrial Fibroblasts

**Figure 2:**
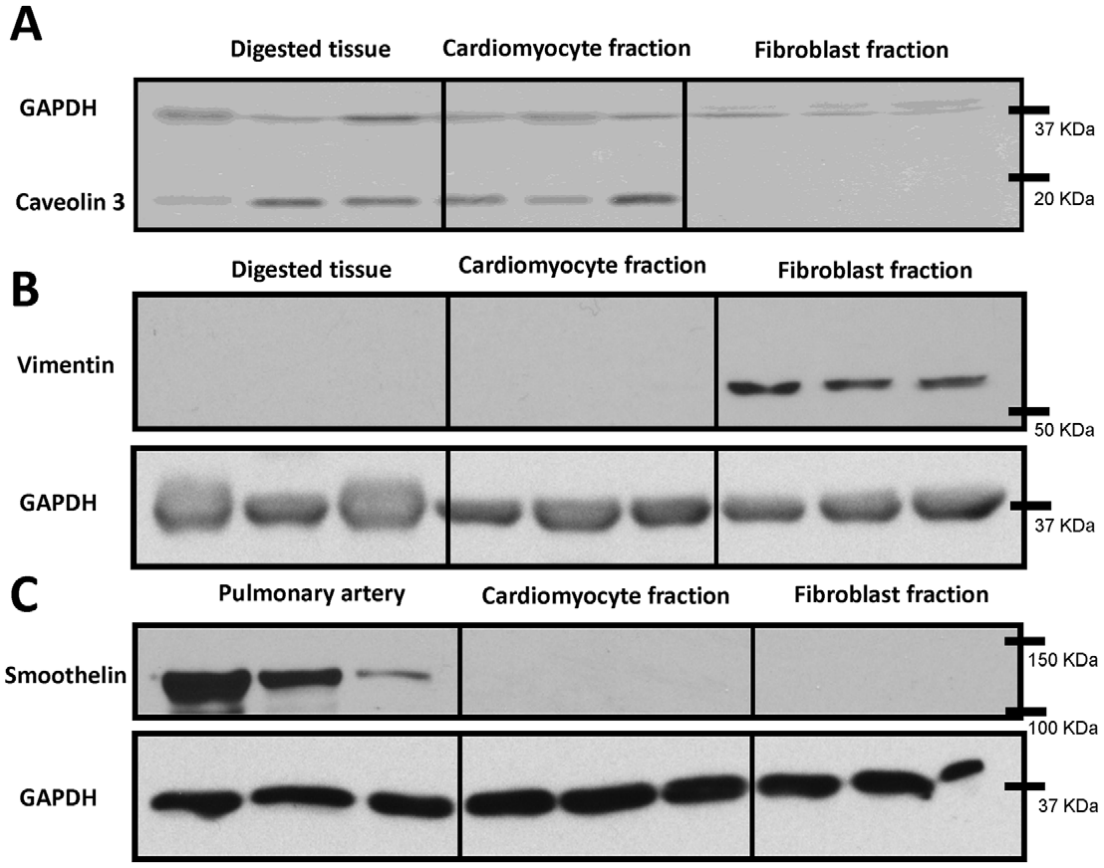
Western blots were used to quantify caveolin 3, vimentin, and smoothelin expression. Caveolin 3 was found in digested whole tissue and also in the cardiomyocyte fraction but was completely absent from the freshly isolated fibroblast fraction (A). Vimentin showed strong expression in the fibroblast fraction but was not seen in whole digested tissue or the cardiomyocyte fraction (B). To assess smooth muscle cell contamination, smoothelin immunopositivity was verified in the cardiomyocyte and fibroblast fractions, and compared to pulmonary artery as a positive control (C). Smoothelin was strongly expressed in pulmonary artery but was not present in the cardiomyocyte or fibroblast fraction. N = 3 dogs/group.

In order to gain a better understanding of fibroblast behaviour between control and CHF dogs, and the discrepancy between tissue gene expression data and fibroblast culture data, freshly isolated fibroblasts were obtained and compared to fibroblasts after 48 hours of culture. First, in order to verify the effectiveness of the fibroblast purification process for freshly isolated fibroblasts, Western blots were performed to assess the presence of potential contaminating cell types. Caveolin 3, a muscle specific protein strongly expressed in cardiomyocytes [15], was used to verify the absence of significant cardiomyocyte contamination in our fresh fibroblast preparation (Figure 2A). Vimentin is expressed in mesenchymal-derived cells, including fibroblasts. Significant vimentin staining was not present in tissue or the isolated cardiomyocyte fraction, but vimentin was highly expressed in the fibroblast fraction (Figure 2B). Lastly, smoothelin expression was examined to evaluate possible smooth muscle cell contamination. Although smoothelin was highly expressed in the positive control (pulmonary artery) it was not present in either the cardiomyocyte or fibroblast fraction (Figure 2C).

**Figure 3:**
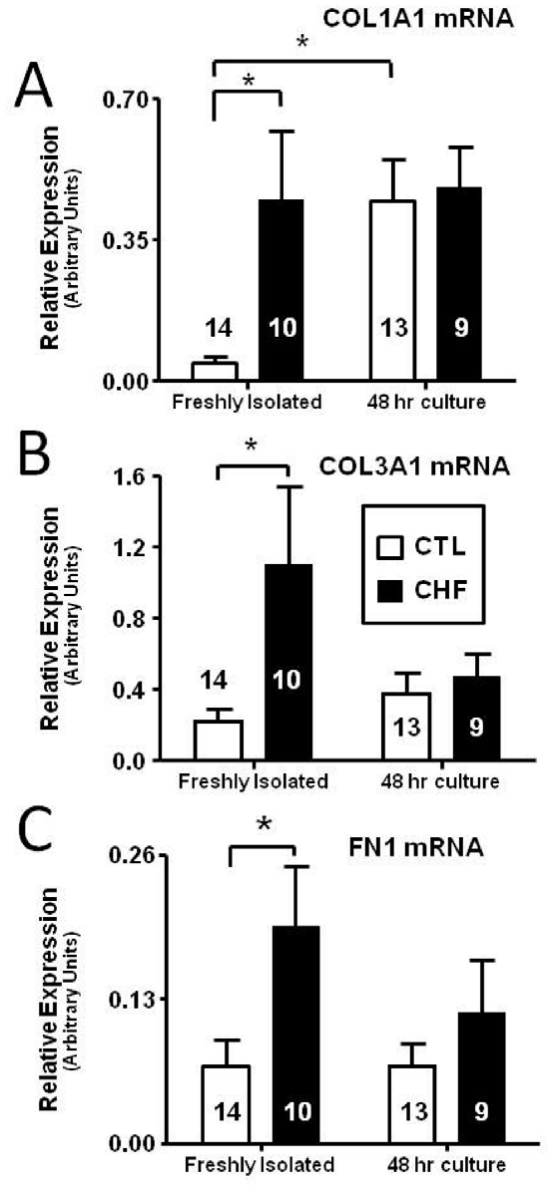
Comparative gene expression studies with freshly isolated versus cultured control (CTL) and CHF dog fibroblasts. (A) COL1A1 (B) COL3A1 (C) Fibronectin (FN1) mRNA expression in freshly isolated CTL and CHF dog fibroblasts and after 48 hour culture. *P<0.05 by ANOVA with Bonferroni post-hoc tests, number of dogs indicated in figure.

The expression of ECM-genes was then assayed in the freshly isolated fibroblast preparation and compared to that in fibroblasts cultured for 48 hours. COL1A1 mRNA expression was strikingly different in freshly isolated control and CHF fibroblasts (Figure 3A, left), consistent with *in vivo* changes. CHF fibroblasts showed an approximately 10-fold greater expression of COL1A1 compared to control fibroblasts. After culture for 48 hours, the mRNA expression of COL1A1 was comparable for the two groups (Figure 3A, right). This change was due to an approximately 10-fold upregulation of COL1A1 gene expression in cultured fibroblasts from control dogs. Whereas CHF gene expression was very strong in freshly isolated fibroblasts, it failed to increase further with culture. Similarly, COL3A1 mRNA showed 5-fold greater expression for freshly isolated CHF fibroblasts versus controls (Figure 3B). After 48 hour culture COL3A1 mRNA expression also became indistinguishable between the two groups, although unlike COL1A1, culture appeared to decrease COL3A1 mRNA expression (Figure 3B). Similar trends were seen for FN1, which showed an approximately 3-fold greater expression in freshly isolated CHF fibroblasts vs. control (Figure 3C). After 48 hours of culture, CHF fibroblasts showed decreased expression of fibronectin, which was no longer significantly different from values in cultured control fibroblasts.

### Time Course of Gene Expression Changes in Culture

**Figure 4:**
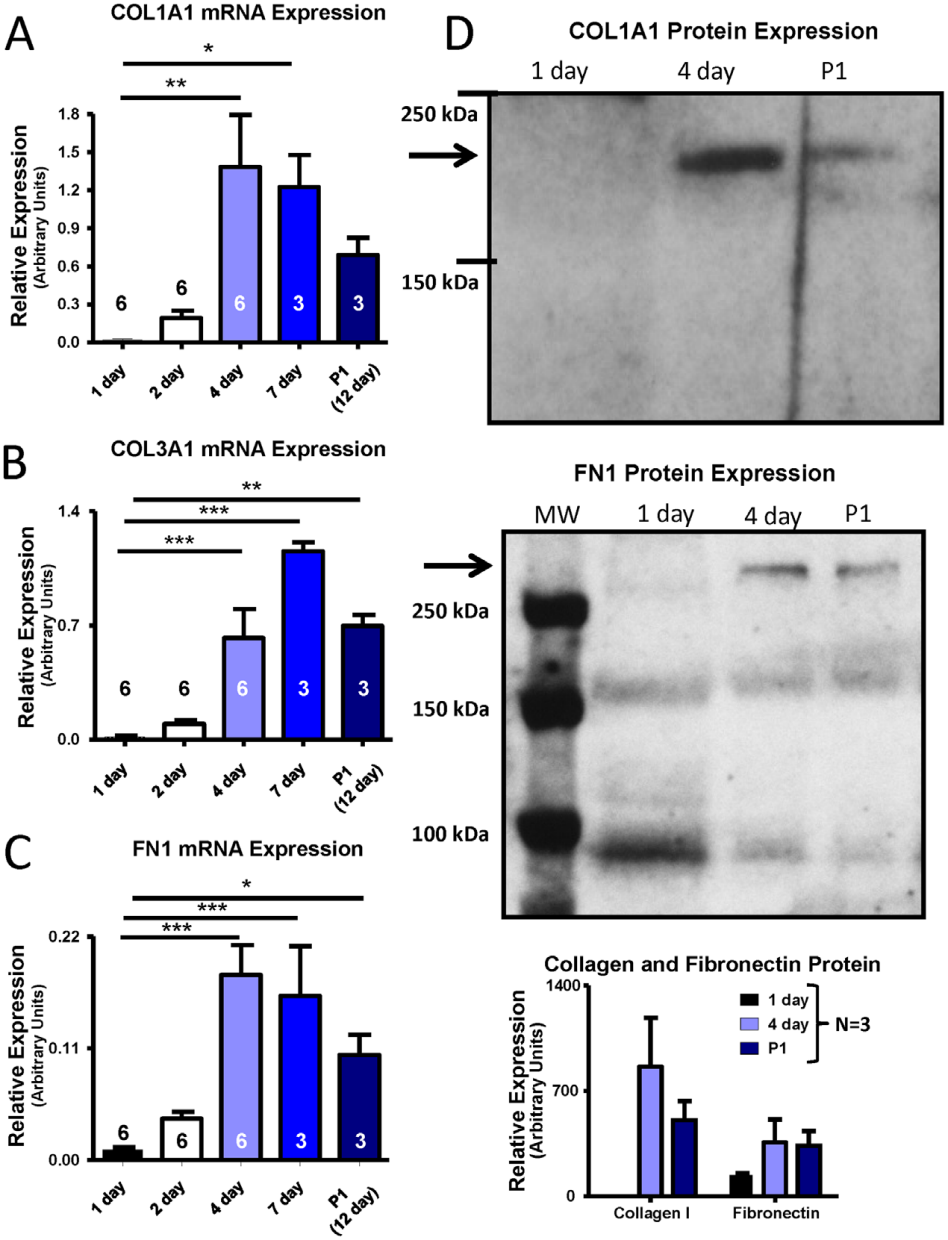
Dependence of fibroblast expression of various ECM proteins on time in culture. Results are shown for (A) COL1A1 mRNA, (B) COL3A1 mRNA, (C) FN1 mRNA, (D) COL1A1 and FN1 protein. Control fibroblasts were cultured for 1, 2, 4, 7, and 12 days. For the last time point, cells were passaged (P1) after 7 days of culture and then grown to confluence (∼5 days). Representative Western blot images for COL1A1 and FN1 are shown in the upper panels of (D), with mean±SEM data beneath. Protein levels were measured from culture supernatant for the 24 hours preceding cell collection. *P<0.05, **P<0.01, ***P<0.001 by ANOVA with Bonferroni post-hoc tests, number of dogs indicated in figure.

We then moved on to assess the time course of gene expression changes in cultured fibroblasts. Fibroblasts were cultured for 24 hours, 48 hours, 4 days (when cell-cell contact has been made), 7 days (first confluence) and 15 days (to allow first confluence, passage, and growth to second confluence) after isolation. The time course of collagen and fibronectin mRNA expression is shown in Figures 4A–C. COL1A1 expression increased progressively from days 1–4, then stabilized to day 7. First-passage (P1) fibroblasts had COL1A1-levels that were slightly lower than at day 7, but were still increased 37-fold as compared to day-1 values (Figure 4A). COL3A1 and FN1 expression showed similar patterns, with levels increased 43-fold and 11-fold respectively at P1 relative to day-1 values (Figures 4B and 4C). FN1 expression peaked between 4 and 7 days of culture (∼18-fold) but did not return to 24 hour culture levels after P1, with expression remaining increased 11-fold vs. 1-day culture. In order to relate the mRNA data to protein-expression changes, Western blots were obtained on cell-culture supernatants and were normalized to cell number. Protein data for collagen-I and FN1 largely paralleled the mRNA results (Figure 4D).

### Morphological Differences in Freshly Isolated versus Cultured Control and CHF Fibroblasts

**Figure 5:**
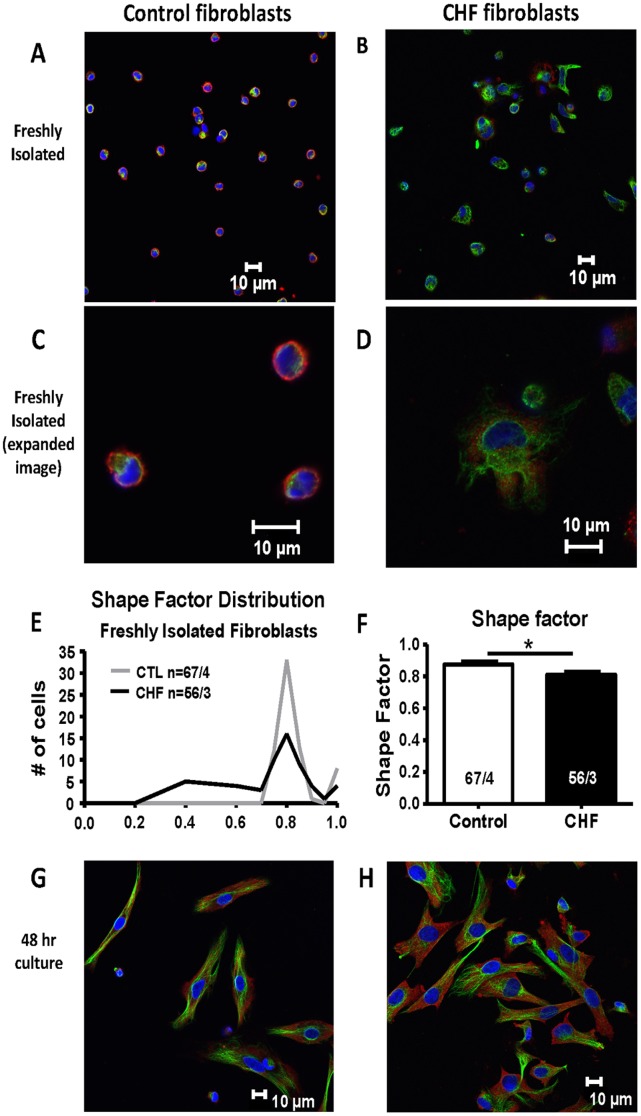
Immunofluorescent images of freshly isolated and cultured fibroblasts. (A) Freshly isolated control (CTL) dog and (B) freshly isolated CHF dog fibroblasts. Expanded images of freshly isolated CTL and CHF fibroblasts are shown in (C) and (D) respectively. Staining shown is DAPI (blue, nuclear), vimentin (green, stress fibers), and α-SMA (red). Shape factor was used to quantify cell spreading in freshly isolated fibroblasts and the distribution is shown in (E). The average shape factor for both CHF and CTL is shown in (F). *P<0.05 by t-test. N/N = Number of cells/number of dogs. (G) and (H), CTL dog (G) and CHF dog (H) fibroblasts after 48 hrs of culture.

After observing differences in gene expression for freshly isolated fibroblasts from control versus CHF dogs, along with the loss of these differences under cell culture conditions, we examined whether other phenotypic differences are present between freshly isolated control and CHF fibroblasts that are lost in culture conditions. Immunoflorescent staining was performed to assess morphological differences between freshly isolated control and CHF fibroblasts (Figure 5A–B). Clear differences in size, staining, and shape were noted. Control fibroblasts were circular and consisted mostly of nuclear staining (shown by DAPI in blue; expanded image in Figure 5C). CHF fibroblasts, on the other hand, were larger by 63% (diameter of 13.4±0.4 µm; 77 cells/3 dogs) compared to control (8.4±0.3 µm diameter; 49 cells/3 dogs; *P*<0.001) and displayed more extensive cytoplasm with discrete pseudopodal elongations and structured vimentin staining (green, Figure 5D). In order to quantify cell-spreading in freshly-isolated fibroblasts, we calculated the shape factor (4πA/P^2^, with A = area and P = perimeter) [16], [17], with values closer to 1 indicating a round phenotype and values closer to 0 indicating a non-round phenotype. The shape factor distributions for control and CHF fibroblasts are shown in Figure 5E. CHF fibroblasts had an average shape factor of 0.81, significantly smaller than control fibroblasts (0.88, *P<0.05, Figure 5F). Although the difference between the group means was relatively small, differences in the distribution of the shape factor were clear (Figure 5E). CHF fibroblasts showed substantial variation in shape, with some cells having extensive projections (shape factors as low as 0.4) while others being more rounded. Control fibroblasts were all round in appearance and had very similar shape factor values close to 1.

After 48-hour culture CHF and control fibroblasts displayed much more similar morphologies, with long spindle-shaped cells and strong vimentin intermediate filament staining (Figure 5G–H). CHF fibroblasts still tended to be slightly larger, but the difference between the two cell types was much less than for fresh cells (average diameter for CHF was 16.9±0.9 µm and for control was 14.0±0.7 µm, *P*<0.05; CHF cell average 18% larger). Thus, the morphological differences between freshly-isolated fibroblasts from CHF-dogs and controls, consistent with greater fibroblast activation in CHF, were attenuated in culture.

### K^+^-channel Expression in Control and CHF Fibroblasts

**Figure 6:**
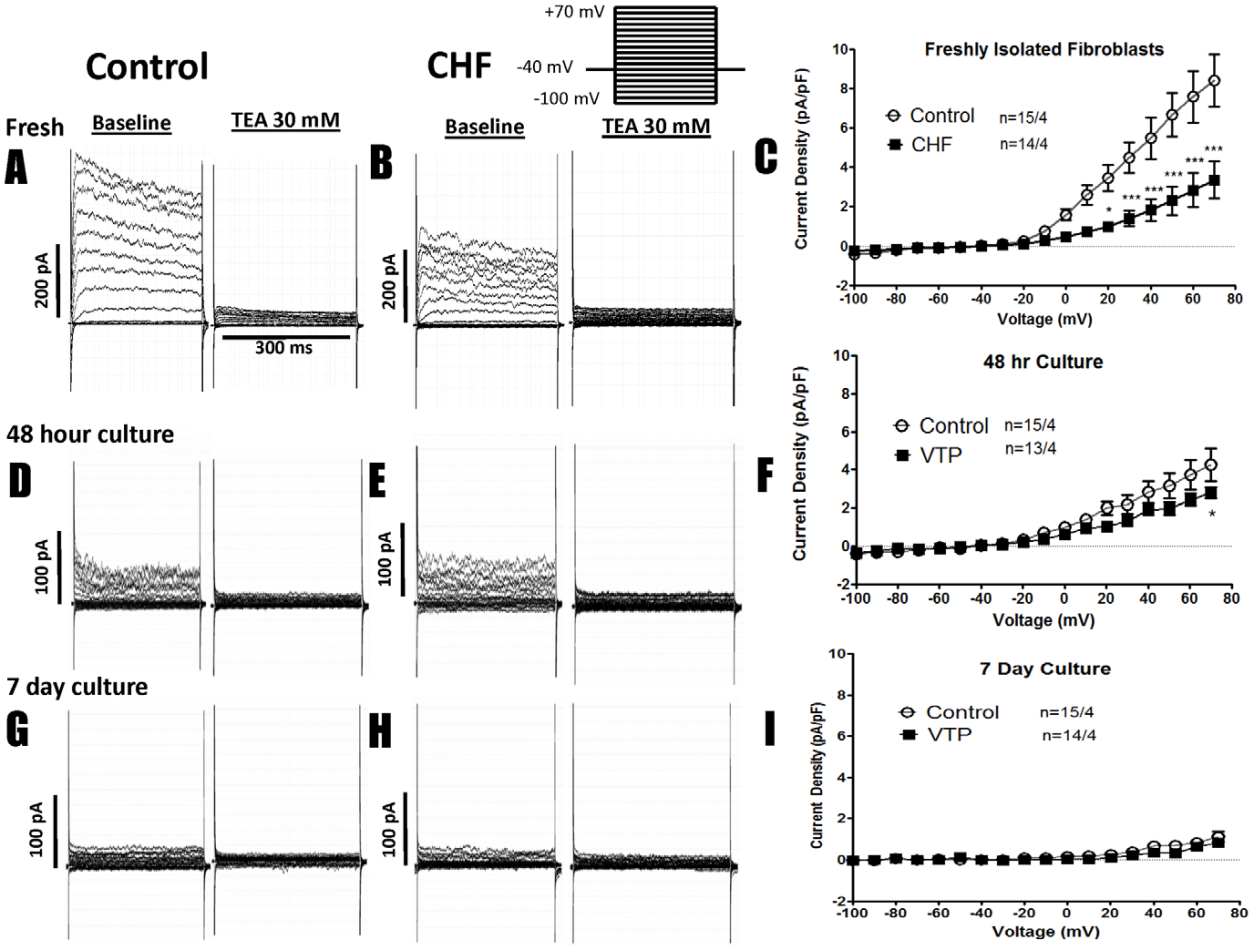
Representative recordings of K^+^-current at baseline and after TEA treatment, along with TEA-sensitive K^+^-current-voltage relations for control and CHF fibroblasts. Results are shown for freshly isolated fibroblasts (top), 48-hour cultured cells (middle) and 7-day cultured cells (bottom). Each set of data shows currents before and after TEA in representative fibroblasts from a control and a CHF dog, followed by mean current-voltage relations. (A,D,G) Recordings from CTL fibroblasts freshly isolated, after 48 hr culture, and after 7 day culture at baseline and after 30 mM TEA. (B,E,H) Recordings from CHF fibroblasts freshly isolated, after 48 hr culture, and after 7 day culture. (C,F,I) The current voltage relationship for control and CHF fibroblasts that were freshly isolated (C), cultured for 48 hours (F), and after culture for 7 days (I). ***P<0.001, *P<0.05 by 2-way ANOVA followed by Bonferroni post-tests. Number of cells studied is indicated first, followed by the number of dogs.

A variety of ionic currents, including K^+^-conductances, have been described in cultured human fibroblasts [18]. To determine whether cell culture alters fibroblast ion currents in our system, TEA-sensitive K^+^-current was first measured in freshly isolated control and CHF fibroblasts (Figure 6A–C). Control fibroblasts displayed substantial currents that were virtually eliminated by 30 mM TEA (representative recordings in Figure 6A), whereas CHF fibroblasts consistently showed much smaller current densities (Figure 6B). Mean current density-voltage relations (Figure 6C) showed substantial and highly-significant decreases in CHF fibroblasts. Overall, control fibroblast current was about 2.5-fold larger than CHF current (e.g. 8.42±1.3 pA/pF in control cells at +70 mV, vs. 3.36±0.9 pA/pF in CHF, *P*<0.001). After 48 hours of culture, control current density decreased by about 50%, so that it was only slightly larger than current in CHF cells (4.27±0.9 pA/pF control vs. 2.79±0.3pA/pF CHF at +70 mV, Figures 6D–F). After 7 days of culture, the K^+^-currents of control and CHF fibroblasts were both strongly downregulated (control current density had decreased by over 85%), and were now indistinguishable from one other (1.1±0.3 pA/pF control vs. 0.9±0.1pA/pF CHF at +70 mV, *P* = NS).

### Potential Role of Culture Surface

**Figure 7:**
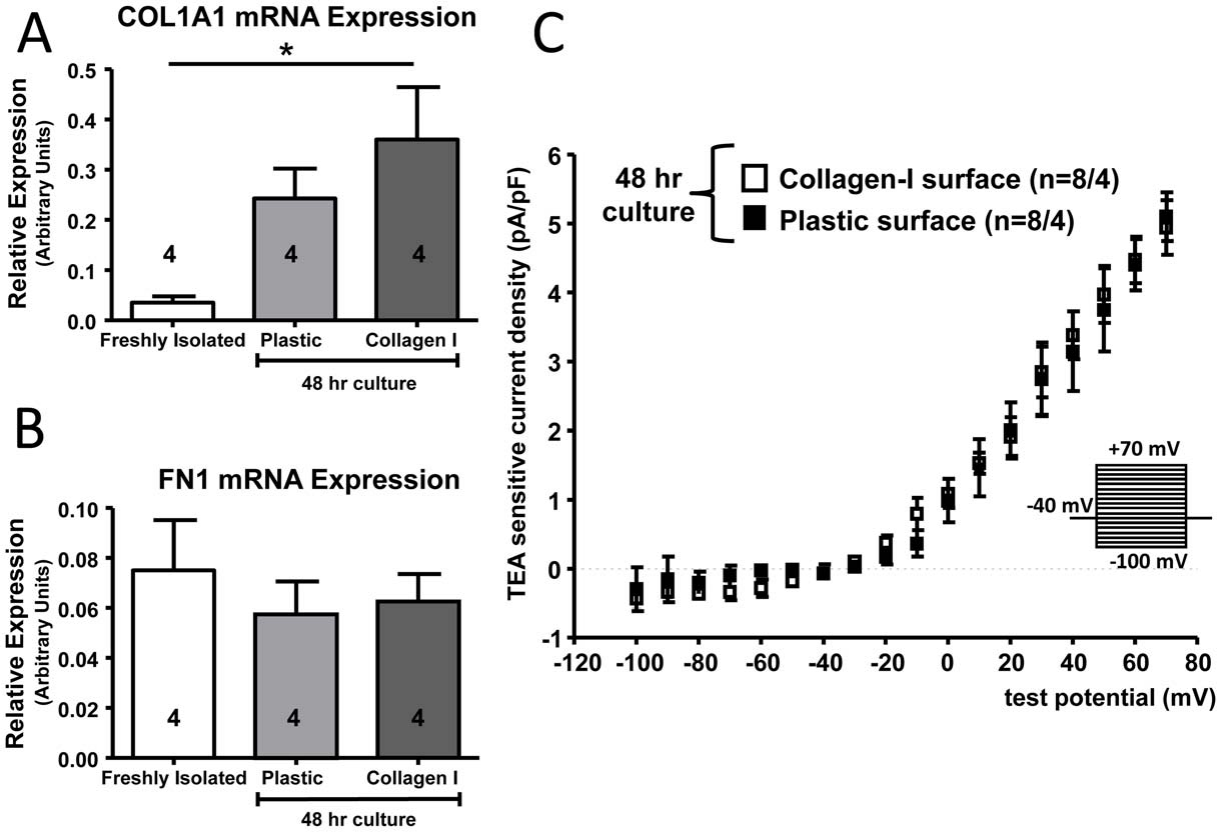
Control fibroblasts were either freshly isolated or grown in culture for 48 hours on standard plastic culture dishes or on plastic culture dishes coated with collagen-I. Gene expression was measured for COL1A1 (A) and FN1 (B). The current-voltage relationship for TEA-sensitive K^+^-current is shown for control fibroblasts grown on plastic and collagen I plates (C). The number of dogs is indicated in the figure, with the patch clamp data showing the number of cells studied/number of animals. *P<0.05 by ANOVA with Bonferroni post-hoc tests.

It is possible that the surface on which fibroblasts are cultured affects their properties. In order to test the hypothesis that hard plastic culture dishes alter fibroblast behaviour, we compared properties of fibroblasts cultured on collagen-I coated plates to those of fibroblasts cultured on non-coated plastic culture dishes. Collagen-I mRNA expression was upregulated in control fibroblasts 48 hours after culture on collagen-I coated surfaces as compared to freshly isolated fibroblasts, to an extent comparable to cells cultured on plastic surfaces (Figure 7A). FN1 gene expression was unchanged across all three groups (Figure 7B). TEA-sensitive K^+^-current was identical for fibroblasts grown on either plastic or collagen-I coated dishes (Figure 7C). Thus, collagen-I coating did not alter the phenotypic properties of cultured fibroblasts.

## Discussion

In this study, we have shown that freshly isolated atrial control and CHF fibroblasts display substantial differences consistent with the observed *in vivo* fibrotic phenotype. Remodeling is manifested at the level of cell morphology, changes in ECM gene expression, and the expression of ion channels. When cells from the same animals are placed in culture, their phenotypic differences are obscured. The phenotypic changes from fresh cells increase over the initial several days of culture and persist after passage.

### Previous Studies of Cultured Fibroblast Phenotypes from Cardiac Remodelled Animals

Flack et al studied the phenotype of cultured ventricular fibroblasts derived from pigs in which CHF was induced by rapid atrial pacing at 240 bpm for 3 weeks [19]. CHF fibroblasts displayed increased migration, adhesion, membrane type-1 matrix metalloproteinase, and vimentin compared to control fibroblasts. The differential phenotype was maintained over 4 passages.

Jarvis et al. investigated myocardial infarct derived (ID) vs. non-infarct derived (NID) ovine ventricular fibroblasts in culture [20]. Myocardial infarction was induced by ligation of the left descending coronary artery and fibroblasts were obtained by migration from infarcted or non-infarcted left-ventricular tissue *in vitro*. Surprisingly, NID fibroblasts expressed higher levels of α-SMA at baseline, whereas ID fibroblasts had higher levels of natriuretic peptide receptor (NPR) A and NPR-B and reacted differently to treatment with TGFβ and PDGF. TGFβ increased NPR-B mRNA levels in NID but decreased levels in ID fibroblasts. Collagen I mRNA expression was also significantly but modestly greater in ID than NID fibroblasts in fibroblasts at passage 4. Neither of these studies examined the properties of freshly isolated fibroblasts, so that attenuation of the phenotype in culture was not assessed. Yperman et al studied changes occurring in cultured interstitial cells (fibroblasts) vs. fresh isolates [21]. In fresh isolates (from all 4 valves) collagen I expression was low, but collagen expression increased in culture at passage 0 (first confluence) and remained stable for 5 passages thereafter.

### CHF-related Atrial Remodeling and Fibroblast Properties

Atrial fibrillation (AF) is the most common cardiac arrhythmia and CHF is a particularly important cause of AF [22]. Atrial fibrosis is an important contributor to the development of AF in the setting of CHF [23]. Fibroblasts may contribute to arrhythmogenesis both by remodeling the ECM to produce tissue fibrosis and via electrical interactions with cardiomyocytes that can promote both focal ectopic activity and re-entry [24], [25]. Atrial fibroblasts are more reactive than ventricular to a variety of stimuli, a property that is associated with greater atrial than ventricular fibrosis over a wide range of pathological contexts [2]. However, the vast majority of previous studies of cardiac fibroblast function have been performed with ventricular fibroblasts. It is therefore particularly important to understand further the determinants of atrial fibroblast function.

### Novel Aspects and Potential Significance

This is, to our knowledge, the first study to evaluate changes in the cellular phenotype of atrial fibroblasts freshly isolated from CHF dogs, and the first to compare in detail the properties of freshly-isolated versus cultured fibroblasts in a cardiac disease model. Freshly-isolated fibroblasts from CHF dogs show evidence of an activated phenotype (altered morphology, increased cell spreading, and increased ECM gene expression) as well as altered K^+^-channel expression. Our results also show that fibroblasts in culture take on many of the properties of disease-remodelled fibroblasts. The activating effect of cell culture conditions on cardiac fibroblasts is not a new observation [26], but what is new is our observation that these changes may obscure and even render undetectable the effects of cardiac disease on atrial fibroblast properties. Thus, caution is required in the interpretation of results from studies of the effects of cardiac remodeling on fibroblast function obtained in cultured cell systems. Correlation with *in vivo* indices and ideally with properties of freshly-isolated fibroblasts is important.

We found it interesting that collagen I and collagen III were differentially regulated in cell culture (Figure 3A/B). Collagen I is the predominant collagen in the heart (∼85%), with collagen III showing less expression (∼11%) [27]; however, this ratio has been shown to change with disease conditions, including AF and CHF [28], [29]. In patients with permanent AF, collagen I is increased, whereas collagen III is not [28]. As cell culture appears to mimic aspects of cardiac disease states associated with tissue-fibrosis (e.g. fibroblasts appear more activated) it is not surprising that collagen is differentially regulated *in vitro* in fashions similar to those previously shown *in vivo*. Thus, collagen-III expression is not significantly altered after 48 hours in culture (Figure 3) and peaks after 7 days of culture (Figure 4), whereas collagen-I expression increases significantly after 48 hours (Figure 3) and appears to peak around 4 days of culture (Figure 4). The early (48-hour) response may parallel acute *in vivo* adjustments, with collagen-I lending more tensile strength whereas collagen III, which imparts resilience, may be part of a longer-term response in some pathologies [27].

Another novel finding in our paper is the downregulation of TEA-sensitive K^+^-channels with cell-culture, mimicking CHF-induced changes. Electrical interactions between cardiomyocytes and fibroblasts play a potentially-important role in cardiac arrhythmogenesis [24], [25] and myofibroblasts have been shown to increase tension on cardiomyocytes, activating mechanosensitive channels and altering conduction [30]. Mathematical models incorporating representations of fibroblast ion channels reproduce a variety of cardiac arrhythmic behaviours, including those occurring in AF [31]–[34]. Changes in fibroblast ion-current phenotype with cell culture and remodeling are therefore potentially important for the understanding of arrhythmia mechanisms in native hearts and model systems. Furthermore, cultured fibroblasts have been used extensively to investigate fibroblast ion-channel expression [18]. Our findings urge caution in the extrapolation of such results to *in vivo* contexts.

### Maintaining the in vivo Phenotype in vitro

Because of the convenience and flexibility of using cultured fibroblasts, it is important to find ways to maintain fibroblast phenotype closer to the *in vivo* state once placed into culture. One approach would be to grow cells on substrates similar to *in vivo* conditions. As collagen-I is the most abundant cardiac ECM protein, we tried to achieve this by growing fibroblasts on plates coated with collagen-I. Unfortunately, we found no detectable differences in gene expression or ionic-current remodeling between fibroblasts grown on non-coated plastic vs. collagen-I coated plates. The stiffness of the substrate has been shown to influence fibroblast behaviour. When stiffness of the substrate was increased incrementally (0.3–55 kPa), lung fibroblasts increased cell numbers at highest stiffness (20–55 kPa) whereas cell numbers were suppressed at lower stiffness (0.3 kPa) [35]. In the normal rat heart, stiffness averages around 18 kPa and can increase ∼3-fold in infarcted hearts [36]. One way to maintain CHF and control fibroblast properties in their respective *in vivo* states despite culture conditions might be to grow them on silicone substrates with low Young’s modulus for control and higher Young’s modulus for CHF fibroblasts. Fibroblasts have been successfully grown on these stiffer substrates; with lower stiffness (∼15 kPa) resulting in decreased cell numbers positive for α-SMA stress fibers after passage 4/5 as compared to a glass substrate [37]. After 12 hours of culture, about 90% of rat embryonic fibroblasts were α-SMA stress-fiber positive at a Young’s modulus ranging from 16–780 kPa, whereas on 9.6 kPa substrates over 97% of cells lost stress fiber formation [38].

### Potential Limitations

It has been shown previously that the specific *in vitro* culture conditions can have an important effect on fibroblast phenotype. For example, culturing human atrial fibroblasts in Dulbecco's Modified Eagle Medium (DMEM) induces a myofibroblast phenotype, whereas the same fibroblasts grown in Endothelial Growth Medium-2 retain the cardiac fibroblast phenotype [39]. This is an additional source of variability that must be considered between and within studies.

### Conclusions

Freshly-isolated atrial fibroblasts from dogs with CHF demonstrate a specific phenotype that is highly-congruent with *in vivo* indices. Culturing control fibroblasts can reproduce many of the phenotypic changes, including morphological alterations, changes in ECM gene expression, and alterations in ion channel expression, that are otherwise caused by CHF, thereby obscuring differences between control and CHF fibroblasts. These considerations are important when attempting to identify cardiac disease-induced changes in fibroblast properties with the use of *in vitro* systems.
